# Regulatory effect of inflammatory mediators in spinal cord injury

**DOI:** 10.3389/fimmu.2025.1619337

**Published:** 2025-08-26

**Authors:** Wen-cong Zeng, Fang-jun Zeng

**Affiliations:** ^1^ Department of Spine Surgery, Ganzhou People's Hospital, Ganzhou, Jiangxi, China; ^2^ Department of Spine Surgery, The Affiliated Ganzhou Hospital of Nanchang University (Ganzhou Hospital-Nanfang Hospital, Southern Medical University), Ganzhou, Jiangxi, China

**Keywords:** spinal cord injury (SCI), inflammatory response, inflammatory mediators, regulatory mechanisms, therapeutic targets

## Abstract

Spinal cord injury (SCI) is a severe disabling central nervous system injury that can lead to severe sensory and motor dysfunction, and even paralysis. Depending on the mechanism of injury, SCI can be divided into primary injury and secondary injury. While secondary injury is the most critical stage in the pathophysiological process of SCI, which is the uncontrolled destructive cascade that follows. At present, symptoms are mainly alleviated and endogenous repair mechanisms are improved through drug intervention, surgical decompression and rehabilitation therapy, but they cannot directly promote nerve regeneration and functional recovery. Recently, an increasing number of studies have shown that the inflammatory response is a core link in secondary injury and plays a crucial role in regulating the pathological progression of acute and chronic SCI. Inflammatory mediators are key participants in the inflammatory response, which can trigger various neuropathological conditions and neurological dysfunction and are related to the severity of the injury. They are being explored as potential therapeutic targets for SCI and related diseases. Therefore, reducing the production of pro-inflammatory mediators is feasible and will also become a research hotspot in the future. This article summarizes the main sources of inflammatory mediators related to injury, their expression regulation, the key signaling pathways that regulate their production (such as Toll-like receptors, NF-κB, MAPK pathways, etc.), and their impact on the pathophysiology of SCI. In addition, treatment methods such as chemical antagonists, plant extracts and hormone therapy have been introduced to inhibit the expression of inflammatory mediators in order to control and improve the inflammatory microenvironment. This article mainly relies on preclinical research evidence to deeply analyze the core position of inflammatory mediators, providing a theoretical basis and direction guidance for the development of more effective SCI anti-inflammatory treatments.

## 1 Introduction

Spinal cord injury (SCI) is a devastating neurological injury that can result in permanent loss of movement and sensation below the injury plane (1, 2). SCI is often caused by accidents such as car accidents, falls from high places and violent impacts (3, 4). The global incidence of SCI is estimated to be between 250,000 and 500,000 people per year (5). The total lifetime medical cost of each SCI patient exceeds $3 million (6), which puts a heavy burden on patients and their families (7). Due to the complex pathophysiological process of SCI and the very weak nerve repair ability, it is difficult for the injured spinal cord to repair and rebuild its function (8, 9). This poses great challenges for healthcare workers and medical research (10, 11). The current treatment strategies for SCI mainly include surgical decompression, hormone shock therapy, drug assistance and rehabilitation therapy (12). Although some progress has been made in surgery and rehabilitation in recent years, there is still a lack of effective cure (13). The pathophysiological process after SCI is extremely complex, involving multiple injury mechanisms. Primary injury is usually caused by external direct violence that causes instantaneous damage to spinal cord tissue, such as spinal cord contusion and fracture, and this process is often difficult to avoid and reverse (14). What is more critical is secondary injury. Within a few minutes to a few weeks after the primary injury, the body will initiate a series of complex reactions, among which inflammation plays a central role in secondary injury and is also an important factor leading to further injury of spinal cord tissue and loss of nerve function (15, 16).

Inflammatory response is a protective mechanism of organisms against internal and external stimuli. Moderate inflammatory response contributes to the stability of the internal environment of the organism, while excessive or sustained inflammation will lead to the occurrence of diseases (17, 18). Sustained inflammatory response is considered to be an important pathological process in the secondary injury stage, which can directly or indirectly determine the outcome of SCI (19, 20). While inflammatory mediators, such as TNF-α, IL-1β and IL-6, have been widely studied and increase rapidly in the early stage of injury. They induce inflammatory response by activating immune cells and changing the permeability of the blood-spinal barrier, leading to injury and apoptosis of neurons and glial cells. Thus hindering the recovery of neurological function (21). In addition, monocyte chemotactic protein-1 (MCP-1) and other chemokines attract immune cells to gather at the injured site, further aggravating the release of inflammatory mediators and inflammatory infiltration, thus triggering an inflammatory cascade (22). This article systematically integrates the main cellular sources of key inflammatory mediators during the secondary injury process after SCI (and their spatiotemporal expression characteristics), clarifying their cascade regulatory effects mediated by the activation of Toll-like receptor/NF-κB/MAPK and other signaling pathways. By targeting and inhibiting the abnormal expression of these mediators, the vicious cycle of the inflammatory microenvironment can be effectively intervened, secondary nerve injury can be alleviated, and conditions for nerve repair can be created. This study not only deepened the understanding of the pathophysiological mechanism of SCI (especially the core link of inflammation-driven progressive injury), but also provided direct theoretical basis and experimental support for the development of precise targeted therapies (such as specific antagonists, multi-target regulation of natural products, and immunomodulatory strategies) It has significant guiding significance for promoting the transformation therapy of SCI from symptom control to nerve regeneration and repair.

## 2 Pathological mechanism of SCI

### 2.1 Primary SCI

The primary injury of SCI is mainly caused by external direct violence, such as traffic accident, height fall, violent impact, etc. (6). These powerful external forces directly act on the spine, resulting in spinal fractures, dislocation and other serious injuries, and then directly damage the spinal cord tissue (23). This primary injury will lead to the interruption of the continuity of spinal cord tissue, the death of nerve cells and the rupture of nerve fibers, and the nerve conduction function will be impaired in an instant (24). Damaged nerve cells cannot transmit nerve signals normally, resulting in limb movement, sensory and autonomic dysfunction below the injury plane, and patients may suffer from limb paralysis, sensory loss, urinary and bowel incontinence and other serious symptoms (25). The extent of primary injury depends on the magnitude, mode of action and injured site of external force, which is often irreversible and brings huge blow to the patient’s body and life (26).

### 2.2 Secondary SCI

**Figure 1:**
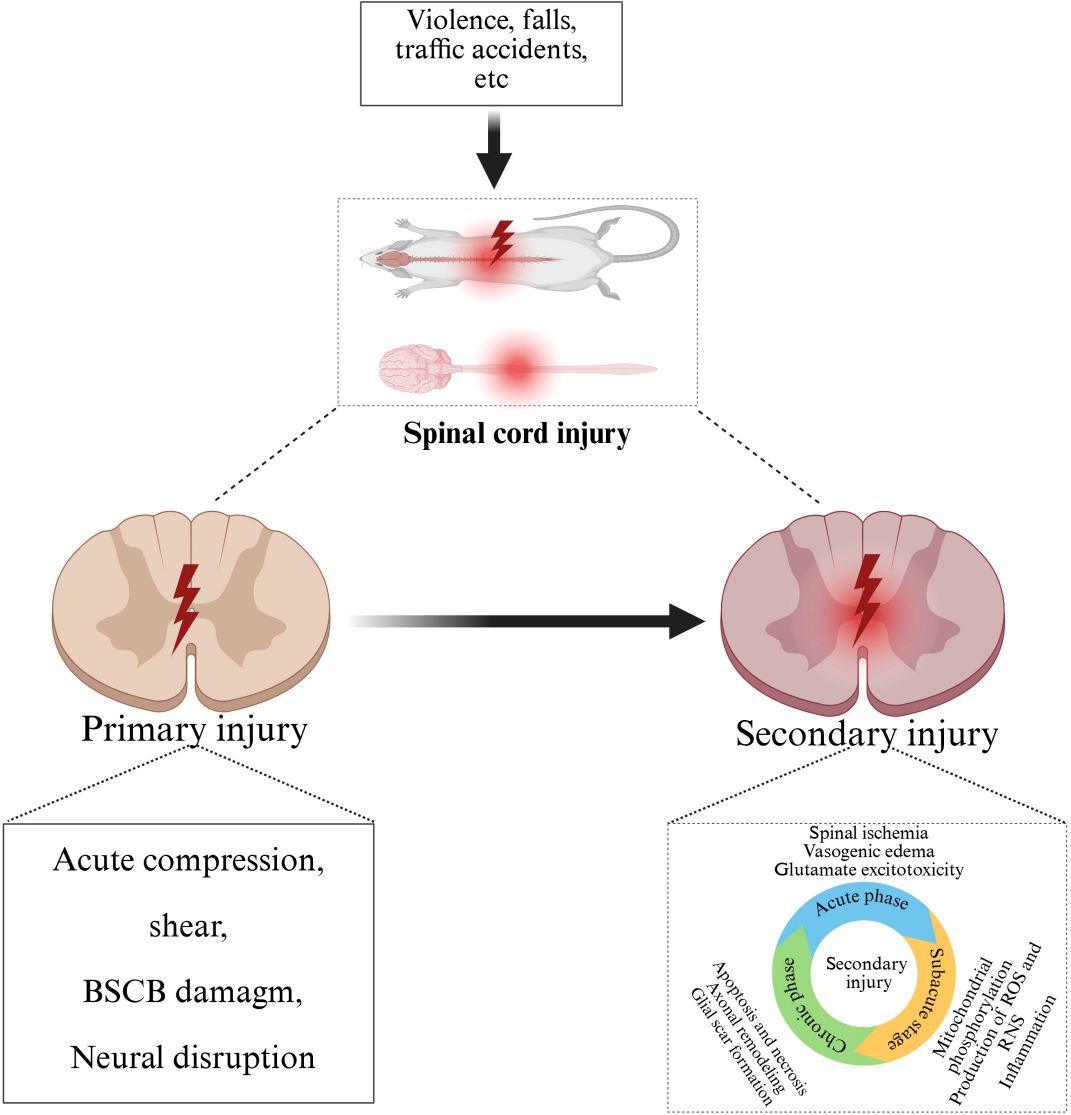
Pathophysiological process after SCI. Traffic accidents, falls, violence and other external forces caused primary SCI, on the basis of primary injury, further development of secondary injury, including acute, subacute and chronic stage.

Secondary injury is a series of complex pathophysiological processes triggered by various factors on the basis of primary injury, which gradually develops within minutes to weeks after SCI, further aggravating the injury of spinal cord tissue and the loss of nerve function (27). It is well known that the most critical stage in the pathophysiological process of SCI involves secondary injury, which is the uncontrolled and destructive cascade that follows. This series of cascade changes mainly include: vascular changes (28, 29), excessive release of excitatory toxins (30), imbalance of ion homeostasis (31, 32), oxidative stress and free radical formation (33, 34), neuronal death (35, 36) and inflammatory response (37–39). Among them, inflammation is the core link of secondary injury. After SCI, nerve cells and glial cells at the injured site release a variety of inflammatory mediators, such as TNF-α, IL-1β, IL-6 and other pro-inflammatory cytokines (40). These inflammatory mediators will activate immune cells, such as macrophages and neutrophils, to aggregate and infiltrate the injured site (31, 41). This leads to the release of more inflammatory mediators and cytotoxic substances, which induces an inflammatory cascade. As the core link of secondary injury (42), inflammatory response not only causes direct damage to the injured spinal cord tissue, but also damages the blood-spinal barrier, triggers apoptosis and necrosis of nerve cells, and affects the repair and regeneration of nerve tissue, thus aggravating the injury of spinal cord tissue (43) (**Figure 1**).

## 3 Source of inflammatory mediators

### 3.1 Secretion of immune cells

Immune cells play a central role in the inflammatory response after SCI, and they are one of the main sources of inflammatory mediators. When the spinal cord is injured, the body’s immune system is rapidly activated, and a variety of immune cells are recruited to the injured site, and a large number of inflammatory mediators are secreted to initiate and regulate the inflammatory response.

Macrophages are one of the earliest activated immune cells in inflammatory response and play a key role in the secretion of inflammatory mediators after SCI (44). In the early stage of injury, monocytes migrate to the injury site through vascular endothelial cells under the action of chemokines, and differentiate into macrophages under the stimulation of the local microenvironment (38). These macrophages can remove dead tissue, pathogens and cell debris from the site of injury, and they also secrete a series of inflammatory mediators, such as TNF-α, IL-1β and IL-6. These cytokines can activate other immune cells and trigger an inflammatory cascade, further aggravating inflammatory damage (45).

Neutrophils are also important sources of inflammatory mediators after SCI. Within a few hours after injury, neutrophils are rapidly recruited to the injury site and migrate to tissues through the space of vascular endothelial cells under the guidance of chemokines (46, 47). Neutrophils congregated at the injured site and released cytokines, chemokines and various proteases, including MMP and neutrophilic elastase, which aggravated tissue edema and necrosis and promoted apoptosis of neurons and oligodendrocytes (48, 49). In addition, neutrophils can also secrete ROS and proteolytic enzymes, which directly damage surrounding nerve cells and tissues (50).

Lymphocytes also play an important role in the inflammatory response after SCI, including T lymphocytes and B lymphocytes (51). T lymphocytes can be divided into different subtypes such as helper T cells (Th), cytotoxic T cells (Tc), and regulatory T cells (TreGs), all of which have different roles in the inflammatory response. Th1 cells mainly secrete pro-inflammatory cytokines such as interferon-γ (IFN-γ), which can enhance the activity of macrophages and promote the development of inflammatory response (52). Th2 cells secrete anti-inflammatory cytokines such as IL-4 and IL-10, which inhibit inflammatory response to a certain extent and promote tissue repair (53). Tc cells can directly kill cells infected by pathogens or abnormal cells, and also secrete some inflammatory mediators to participate in the regulation of inflammatory response (54). After being stimulated by antigen, B lymphocytes will differentiate into plasma cells to produce antibodies, thus participating in humoral immune response (55). In the case of SCI, B lymphocytes may indirectly participate in the regulation of inflammatory response by secreting cytokines (56).

### 3.2 Release of glial cells

In the inflammatory response after SCI, glial cells not only participate in maintaining the stability of neural microenvironment, but also play an important role in the inflammatory process by releasing inflammatory mediators. After SCI, astrocytes and microglia are rapidly activated and become important sources of inflammatory mediators.

Astrocytes are the most abundant glial cells in the spinal cord, and they undergo significant morphological and functional changes after SCI (57). Under the stimulation of injury, the astrocytes enlarged their cell bodies, increased and thickened their protrusion, and showed a highly reactive state. Activated astrocytes can release a variety of inflammatory mediators, including TNF-α, IL-1β, IL-6, etc. These pro-inflammatory cytokines can further activate immune cells and attract them to gather and infiltrate the injured site, thus exacerbating the inflammatory response (58). In addition, reactive astrocytes can also secrete arachidonic acid metabolites such as prostaglandins, which can regulate vascular permeability and lead to local edema, further exacerbating spinal cord tissue compression and ischemia (59).

As the inherent immune cells of the central nervous system, microglia are also rapidly activated and release inflammatory mediators after SCI (60). In the early stage of injury, microglia mainly polarize to M1 type and become an important source of pro-inflammatory cells. M1-type microglia secrete a large amount of pro-inflammatory mediators and neurotoxic molecules such as TNF-α, IL-1β, IL-6 and NO, which can trigger a strong inflammatory response and cause damage to nerve tissue (61, 62). With the passage of time, some microglia will become polarized towards M2 type, and M2 type microglia mainly secrete anti-inflammatory cytokines, such as IL-10 and TGF-β, which can inhibit inflammation and promote neuroprotection and tissue repair (63). However, in the pathological state after SCI, the polarization balance of microglia is often broken, and the over-activation of M1 microglia and the shortage of M2 microglia make it difficult to control the inflammatory response effectively, thus aggravating the injury of spinal cord tissue (64).

### 3.3 Damaged nerve cells

After SCI, damaged nerve cells themselves produce and release a variety of inflammatory mediators, which play an important role in the inflammatory response and secondary injury after SCI. When a nerve cell is damaged, its integrity is damaged and substances inside the cell are released into the extracellular environment. At the same time, the metabolic and signal transduction pathways of cells are also disturbed, and these changes will cause nerve cells to produce a series of inflammatory mediators, such as IL-1β and TNF-α (65). IL-1β can activate surrounding immune cells and glial cells, prompting them to release more inflammatory mediators, thus triggering an inflammatory cascade (66). While TNF-α can directly induce apoptosis of nerve cells and glial cells, and at the same time increase vascular permeability, leading to spinal cord edema and further aggravate spinal cord tissue injury (67). Damaged nerve cells also produce NO and other inflammatory mediators. After SCI, the expression of inducible nitric oxide synthase (iNOS) is up-regulated, resulting in an increase in the production of NO in nerve cells. Excess NO will combine with superoxide anion to form strong oxidizing nitrite peroxide, resulting in oxidative damage to cells and tissues. In addition, NO can also activate the apoptosis signaling pathway, induce the apoptosis of nerve cells and glial cells, and further aggravate the injury of spinal cord tissue (68).

**Figure 2:**
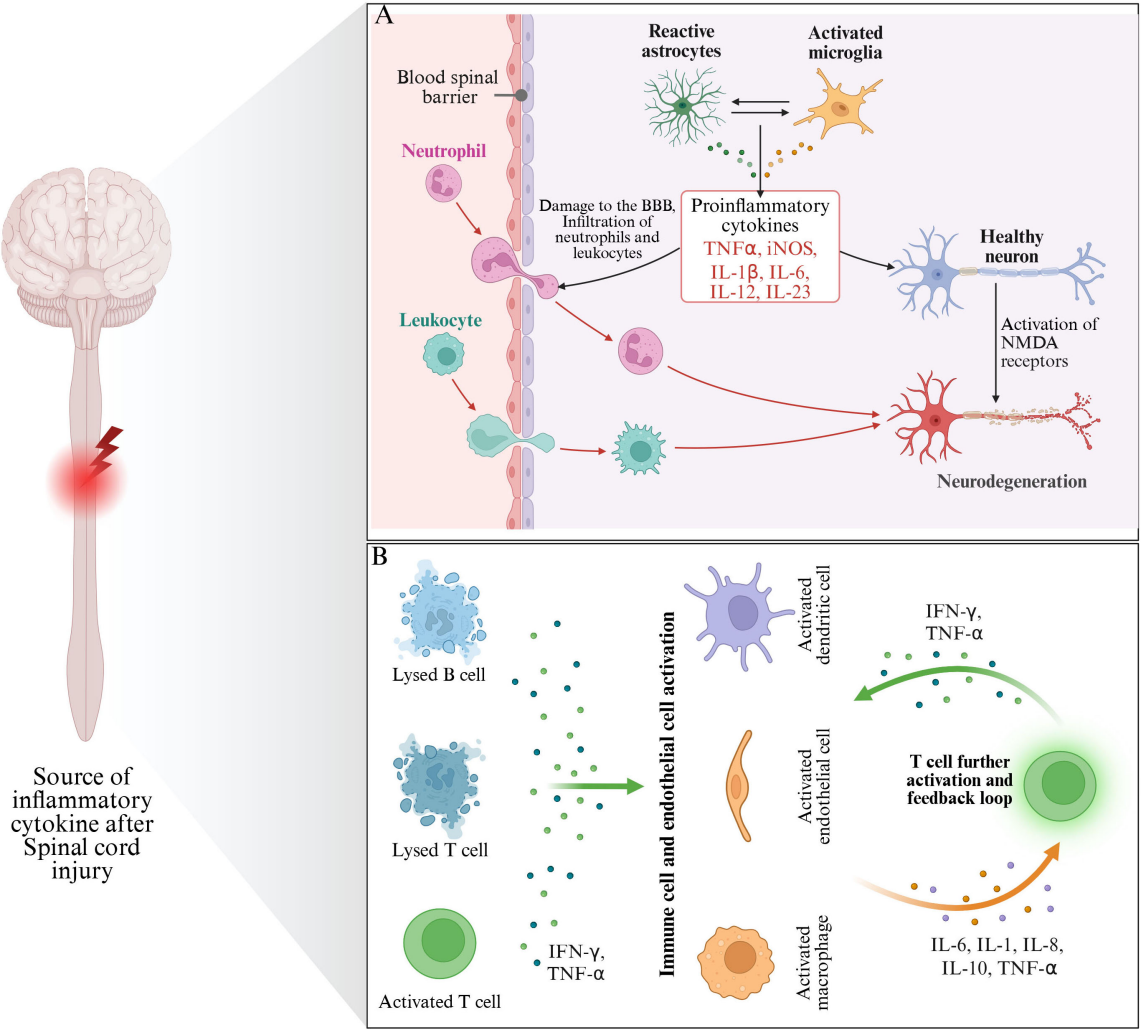
The main source of inflammatory mediators after SCI. **(A)** Indicates that inflammatory mediators are mainly derived from glial cells and nerve cells after injury. **(B)** Indicates that the inflammatory mediators mainly originate from immune cells (mainly including Lysed B cells, Lysed T cells and activated T cells).

In addition to the above inflammatory mediators, damaged nerve cells may also release some other bioactive substances, such as prostaglandins and other arachidonic acid metabolites. These substances can participate in the regulation of inflammatory response by regulating vasomotor, cell proliferation and differentiation (69). Prostaglandin E2 (PGE2) can dilate blood vessels, increase vascular permeability, lead to plasma exudation and local edema, and further aggravate spinal cord tissue compression and ischemia (70). In addition, PGE2 can also regulate the function of immune cells and promote the activation of inflammatory cells and the release of inflammatory mediators, thus exacerbating the inflammatory response (71) (**Figure 2**).

## 4 Classification and function of inflammatory mediators

### 4.1 Cytokines are inflammatory mediators

Cytokines are a kind of small molecular proteins with extensive biological activities synthesized and secreted by immune cells and some non-immune cells after stimulation, and play a central role in the inflammatory response after SCI. Among them, cytokines such as the interleukin (IL) family and tumor necrosis factor (TNF) have significant pro-inflammatory and anti-inflammatory effects (**Table 1**).

**Table 1:** Cytokines and chemokines involved in inflammation after SCI.

Name	Source cell	Function in SCI	References
IL-1β	Monocytes; macrophages	Enhance the inflammatory response; Aggravating oxidative stress	(72, 73)
IL-4	T cells; macrophages	Increased arginase expression and decreased inflammatory mediators	(74, 75)
IL-6	T cells; macrophages	Enhance inflammatory response and promote neuronal apoptosis	(63)
IL-8	Neutrophil	Enhance the inflammatory response; Induced angiogenesis	(76)
IL-9	Th2 cell	Amplify inflammatory responses	(77)
IL-17	CD4^+^ T cell, Th17 cell	Amplify inflammatory responses	(78, 79)
TNF-α	Neutrophils; Macrophage; T cells; B cell	Enhance the inflammatory response; Promote apoptosis and autophagy	(31, 80)
MCP-1	Monocyte	Binds to the CCR2 receptor and promotes the release of inflammatory mediators	(81)
Prostaglandin	Monocyte, macrophage	Increases vascular permeability, leads to plasma exudation and tissue edema	(82)
Leukotriene B4	Neutrophil	Activate neutrophils to release lysosomal enzymes, reactive oxygen species and other toxic substances, causing vasoconstriction	(83, 84)
Histamine	Basophilic granulocyte	Promote the chemotaxis and activation of inflammatory cells, further aggravating the inflammatory response	(85)
5-HT	——	Stimulate macrophages to secrete IL-1, IL-6 and enhance the intensity of inflammatory response	(86)

The interleukin (IL) family mainly includes IL-1 (IL-1α and IL-1β), IL-4, IL-6, IL-8, IL-9, IL-10, IL-17 and so on. IL-1 is one of the earliest discovered proinflammatory cytokines, mainly produced by mononuclear macrophages, endothelial cells, and fibroblasts (87). IL-1 is released rapidly after SCI and can activate immune cells to produce more inflammatory mediators, such as IL-6 and TNF-α, thereby amplifying the inflammatory response (88). IL-1 can also stimulate the synthesis of prostaglandin and nitric oxide, leading to vasodilation and increased permeability, causing spinal cord tissue edema, and further aggravating SCI (89). In addition, IL-1 can also induce nerve cell apoptosis by activating apoptosis-related proteins, destroying the normal structure and function of nerve cells, and hindering the recovery of nerve function (90).

IL-4 is mainly produced by immune cells, but its receptors are found in various cell types and regulate the activity of various immune cells (91). In SCI, IL-4 can drive M2-type macrophages and may support some degree of endogenous repair (92). Meanwhile, IL-4-treated macrophage culture medium enhanced neurite growth *in vitro* (93). In addition, IL-4 increased arginase expression and decreased inflammatory mediators, including IL-1β and TNF-α (74, 75).

IL-6 is a pleiotropic cytokine that can be secreted by monocytes, macrophages, T cells and B cells. IL-6 plays an important role in the inflammatory response after SCI. In the early stage of SCI, the expression of IL-6 increases rapidly, and its main function is to promote the synthesis of acute phase protein, enhance the activity of immune cells, and mediate the cascade amplification of inflammatory response. IL-6 can also promote the proliferation and differentiation of T cells and B cells, and regulate the immune response. In the microenvironment after SCI, high levels of IL-6 can lead to overactivation of inflammatory responses, resulting in toxic effects on nerve cells, affecting nerve cell survival and recovery of nerve function. IL-6 can promote the proliferation of glial cells by activating the signal transduction and transcriptional activator 3 (STAT3) signaling pathway, forming glial scar and hindering the regeneration of nerve axons.

IL-9 is a polymorphic cytokine that regulates Th2 inflammatory response (94). Studies have found that IL-9 is overproduced in the acute phase of SCI, and excess IL-9 can promote the inflammatory response of injury (95). IL-10 is an important anti-inflammatory cytokine, which is mainly produced by mononuclear macrophages, T cells, B cells and activated astrocytes (96, 97). After SCI, the expression of IL-10 is up-regulated, which can inhibit the production of pro-inflammatory cytokines such as IL-1, IL-6, TNF-α, etc., and reduce the release of inflammatory mediators, thus reducing the injury of inflammatory response to spinal cord tissue (98). IL-10 can also regulate the activity of immune cells, inhibit the activation of macrophages and the secretion of inflammatory factors, and promote the production of anti-inflammatory cytokines, such as transforming growth factor-β (TGF-β) (99). By inhibiting the inflammatory response, IL-10 helps to maintain the stability of the microenvironment of the spinal cord tissue, reduce the damage of nerve cells, and promote the recovery of nerve function. However, when secondary damage storms strike, the supply of endogenous IL-10 is insufficient to repair the body (100). Therefore, in the treatment of SCI, exogenous supplementation of IL-10 may improve nerve function by reducing inflammatory response and inhibiting secondary injury, limiting the damage of SCI (99, 101).

IL-17 is a cytokine, mainly produced by the T helper cell 17 subgroup of CD4+ T cells, which plays a crucial role in various inflammatory diseases (102). It was found that the expression of IL-17 in CD4 and CD8 lymphocytes increased to varying degrees after SCI. IL-17 has been shown to be involved in neuroinflammatory processes and is associated in the body with severe stages of disease (103). In addition, IL-17 appears to negatively regulate the regeneration process of ependymal cells after SCI, thus impairs recovery (104).

TNF-α is a proinflammatory cytokine with a wide range of biological activities, mainly produced by activated mononuclear macrophages (105). After SCI, a large amount of TNF-α is released, which can directly damage nerve cells and vascular endothelial cells, increase the permeability of the blood-spinal barrier, lead to the exudation of inflammatory cells and plasma proteins, and cause spinal cord edema (106). TNF-α can also activate neutrophils and macrophages to release more inflammatory mediators and cytotoxic substances, such as ROS and NO, and further aggravate spinal cord tissue injury (107). TNF-α can activate intracellular apoptosis signaling pathway by binding to receptors on the surface of nerve cells, leading to apoptosis of nerve cells (108). In animal models of SCI, inhibiting the expression or activity of TNF-α can significantly reduce inflammatory response, reduce nerve cell apoptosis, and improve nerve function (109).

### 4.2 Chemotactic agents are inflammatory mediators

Chemokines are a class of small molecular proteins that can attract the directed migration of immune cells and play a key regulatory role in inflammatory response (110). By binding to specific receptors on the surface of immune cells, they guide immune cells to gather at the site of inflammation, thus participating in the occurrence and development of inflammatory response. After SCI, the expression of chemokines is rapidly upregulated, attracting a variety of immune cells to migrate to the injury site, further aggravating the inflammatory response.

Monocyte chemotactic protein-1 (MCP-1) is an important member of the chemokine family (111). MCP-1 can bind to CCR2 receptors on the surface of monocytes and macrophages, and then attract these cells to chemotaxis to the lesion site, thus promoting the release of inflammatory mediators (81). Studies have found that after SCI, nerve cells, glial cells and endothelial cells at the injured site secrete a large amount of MCP-1 (112).

IL-8 is a chemokine with unique CXC amino acid sequence, which has a strong chemotactic effect on neutrophils (113). In the early stage of SCI, the expression of IL-8 increases rapidly and is mainly produced by monocytes, macrophages, endothelial cells and epithelial cells (114). IL-8 binds to CXCR1 and CXCR2 receptors on the surface of neutrophils to guide neutrophils to rapidly gather at the site of injury and release a large amount of proteolytic enzymes, reactive oxygen species and other toxic substances, causing damage to surrounding nerve cells and tissues, thus further aggravating the inflammatory response and tissue destruction after injury (115, 116).

### 4.3 Arachidonic acid metabolites

Arachidonic acid (AA) is a polyunsaturated fatty acid that is abundant in cell membrane phospholipids (117). When the cell is stimulated, phospholipase A2 on the cell membrane is activated, and hydrolyzed phospholipids release arachidonic acid. Under the action of a series of enzymes, arachidonic acid metabolizes to produce a variety of bioactive products, including prostaglandins (PG) and leukotrienes (LT), etc. (118, 119). These metabolites play an important role in the inflammatory response after SCI, with extensive effects on blood vessels and cells.

Prostaglandins are a kind of lipid mediator produced by arachidonic acid metabolism through the cycoperoxidase (COX) pathway, including prostaglandin E2 (PGE2), prostaglandin F2α (PGF2α), prostaglandin I2 (PGI2) and other subtypes (120). After SCI, the synthesis and release of prostaglandins in local tissues are significantly increased (70). PGE2 is a biologically important prostaglandin, which can cause vasodilation, increase vascular permeability, lead to plasma exudation and tissue edema. In addition, PGE2 can also increase the sensitivity of pain receptors, resulting in increased pain (82).

Leukotrienes are another important inflammatory mediators produced by arachidonic acid metabolism through 5-lipoxygen (5-LOX) pathway, mainly including leukotriene B4 (LTB4), leukotriene C4 (LTC4), leukotriene D4 (LTD4) and leukotriene E4 (LTE4) etc. (121). Among them, LTB4 is a potent neutrophil chemokine, which can attract a large number of neutrophils to gather at the site of inflammation, and enhance the infiltration of inflammatory cells and the intensity of inflammatory response (122). LTB4 can also activate neutrophils to release lysosomal enzymes, reactive oxygen species and other toxic substances, causing damage to surrounding tissue cells (83, 84). LTC4, LTD4 and LTE4 mainly caused vasoconstriction, bronchospasm and increased vascular permeability, leading to tissue ischemia, hypoxia and edema.

### 4.4 Other inflammatory mediators

In addition to the above major inflammatory mediators, histamine, 5-hydroxyserotonin (5-HT) and other bioactive substances also play an important role in the inflammatory response after SCI. Histamine is a biogenic amine that is widely found in the body and is mainly released by mast cells and basophils. After SCI, local tissue damage and inflammatory stimulation can cause mast cell degranulation and release a large amount of histamine (123). Histamine has a variety of biological effects, it can dilate blood vessels, increase vascular permeability, lead to plasma exudation and tissue edema. Histamine can also stimulate nerve endings, causing itching and painful sensations (124). In the inflammatory response of SCI, histamine exerts its biological role by binding to histamine receptors on the surface of vascular endothelial cells and nerve cells. Histamine binds to H1 receptors on vascular endothelial cells to activate phospholipase C, resulting in increased intracellular calcium ion concentration, vascular smooth muscle relaxation and increased vascular permeability. Histamine can also promote the chemotaxis and activation of inflammatory cells, further aggravating the inflammatory response (85).

5-HT is an important neurotransmitter and inflammatory mediator, and its synthesis and release increase after SCI (125). 5-HT can regulate the contraction and relaxation of blood vessels and affect local blood circulation. In inflammatory response, 5-HT can promote platelet aggregation and thrombosis, increase vascular permeability, and lead to exudation of inflammatory cells and tissue edema (125). 5-HT can also bind to receptors on the surface of immune cells, regulate the activity of immune cells, and promote the release of inflammatory mediators (126). 5-HT can stimulate macrophages to secrete IL-1, IL-6 and other pro-inflammatory cytokines, and enhance the intensity of inflammatory response. 5-HT can also affect the function of nerve cells and participate in the regulation of pain and the repair process of nerve injury (86).

### 4.5 The interaction between different types of inflammatory mediators

#### 4.5.1 Synergistic effect

Recent studies have revealed that there exists a superadditive effect among multiple media, meaning that the combined effect of multiple media far exceeds the sum of the effects of a single medium (127). For example, the synergy of IL-1β and TNF-α can significantly amplify the expression levels of IL-6 and IL-8. This synergistic effect causes the inflammatory response to exhibit nonlinear amplification characteristics, explaining why local injury can lead to extensive tissue destruction (128). Meanwhile, IL-1β and TNF-α form a positive feedback loop: TNF-α enhances the processing and release of IL-1β precursors, while mature IL-1β further promotes the synthesis of TNF-α (129). This positive feedback mechanism leads to a rapid escalation of inflammatory responses after SCI. In addition, IL-1β can also significantly enhance the effect of chemokines. Experimental studies have shown that IL-1β pretreatment can enhance the reactivity of vascular endothelial cells to MCP-1 and promote the crossing of monocytes/macrophages across the blood-spinal cord barrier (BSCB) (130). Furthermore, the synergistic effect of IL-1β and IL-17 deserves attention: At the infiltration site of Th17 cells, IL-1β can enhance the expression of IL-17 receptors, and IL-17 can amplify the production of CCL20 and IL-8 induced by IL-1β, forming a pro-inflammatory microenvironment (131).

Chemokines exhibit a cooperative gradient guidance phenomenon during the recruitment of immune cells. MCP-1(CCL2) and IL-8(CXCL8) form a complementary chemical gradient after spinal cord injury: MCP-1 mainly recruits monocytes/macrophages, while IL-8 specifically attracts neutrophils (132, 133). The study also found that there is an interactive enhancement relationship between these two chemokines -MCP-1 stimulates macrophages to release IL-8, and IL-8 in turn promotes neutrophils to release MMP-9, which releases more latent MCP-1 by degrading the extracellular matrix (134).

The cross-pathway synergy in the arachidonic acid metabolic network is an important mechanism for inflammatory amplification. There is a close interaction between PGE2 produced by the COX-2 pathway and LTB4 produced by the 5-LOX pathway: PGE2 enhances the activity of 5-LOX by increasing the intracellular cAMP level and promotes the synthesis of LTB4. LTB4 amplifies the expression of COX-2 by activating the PKC signal (135, 136). This metabolic cascade leads to excessive aggregation of neutrophils at the site of injury. Research has found that prostaglandins form a bidirectional enhancement loop with cytokines: PGE2 stimulates macrophages to produce IL-23 through the EP4 receptor, which in turn promotes the differentiation of Th17 cells and their secretion of IL-17. IL-17 can induce various cells to express COX-2 and increase the synthesis of PGE2 (38, 137). This circuit enables the inflammatory response to persist after SCI.

#### 4.5.2 Antagonistic effect

In addition to the synergistic effect, there are also antagonistic relationships among different inflammatory mediators. For instance, the balance between IL-4 and IL-9 in Th2 immunity is particularly typical: IL-4 inhibits the differentiation of Th9 cells and reduces the production of IL-9; IL-9 promotes its own secretion by enhancing the expression of PU.1, while inhibiting IL-4-mediated macrophage polarization. In the SCI microenvironment, overexpression of IL-9 can block the protective effect of IL-4 and form a pro-inflammatory state (138). In the chemokine network, receptor competitive antagonism is an important regulatory mechanism. CXCL12 shares the CXCR4 receptor with CXCL10, but they perform opposite functions: CXCL12 promotes neural repair, while CXCL10 exacerbates inflammatory damage (139). The latest research has found that exogenous administration of CXCL12 after SCI can competitively inhibit the damaging effect of CXCL10 and promote the regeneration of nerve axons. In addition, there is also antagonism among lipid mediators. Anti-inflammatory lipid mediators such as resolvin and lipoxin can antagonize the effects of pro-inflammatory leukotrienes (140). Lipoxin A4 inhibits LTB4-induced neutrophil chemotaxis and activation by binding to ALX/FPR2 receptors, promoting inflammation resolution (141). The disruption of this endogenous lipid mediator balance may be an important factor for the persistence of inflammation after SCI.

## 5 Regulation of inflammatory mediators in SCI

### 5.1 Initiate inflammatory response

After SCI, the release of inflammatory mediators is a rapid and complex process. Under the stimulation of primary injury, damaged nerve cells, glial cells and infiltrated immune cells will immediately release a variety of inflammatory mediators, thus initiating inflammatory response (142). TNF-α as one of the earliest released inflammatory mediators, is mainly produced by activated mononuclear macrophages. TNF-α can not only directly cause damage to nerve cells and vascular endothelial cells, increase the permeability of the blood-spinal barrier, but also act as a powerful signaling molecule to activate other cells to release more inflammatory mediators (143). Il-1β binds to the IL-1 receptor on the cell surface and activates a series of intracellular signaling pathways, such as the nuclear transcription factor-κB (NF-κB) signaling pathway and mitogen-activated protein kinase (MAPK) signaling pathway. The activation of these signaling pathways leads to the up-regulation of the expression of multiple genes, including genes of other inflammatory mediators, thus triggering the cascade release of inflammatory mediators (144).

Chemokines also play an important role in the mutual activation of inflammatory mediators. After SCI, MCP-1 is released in large quantities, which can attract monocytes, macrophages and other immune cells to gather at the injured site. After arriving at the injury site, these aggregated immune cells will be activated by the local inflammatory microenvironment and release a large number of inflammatory mediators, such as cytokines and reactive oxygen species, which further aggravate the inflammatory response and lead to the aggravation of spinal cord tissue injury (145). Studies have shown that inhibiting MCP-1 expression or blocking its binding to receptors in animal models of SCI can reduce monocyte and macrophage infiltration and reduce inflammatory response, thereby improving nerve function (146).

Arachidonic acid metabolites, such as prostaglandins and leukotrienes, also play an important role in the release and mutual activation of inflammatory mediators. In the inflammatory response of SCI, PGE2 regulates the activity of immune cells by binding to receptors on the surface of immune cells, promotes the release of inflammatory mediators, and further aggravates the inflammatory response. PGE2 can promote the secretion of IL-1, IL-6 and other pro-inflammatory cytokines by macrophages, and enhance the intensity of inflammatory response (71, 147).

### 5.2 Induced apoptosis

In the complex pathophysiological process after SCI, inflammatory mediators such as TNF-α and IL-1β play a key role, which can induce nerve cell apoptosis through various ways and seriously affect the recovery of nerve function (148). TNF-α binds specifically to tumor necrosis factor receptor 1 (TNFR1) on the surface of nerve cells, and this binding causes TNFR1 to form a trimer structure. Trimerized TNFR1 recruits a series of downstream signaling proteins through its intracellular cysteine-rich death domain (DD), Such as tumor necrosis factor receptor-associated death domain protein (TRADD), Fas associated death domain protein (FADD), tumor necrosis factor receptor-associated factor 2 (TRAF2) and receptor interaction protein (RIP), etc., form death inducing signaling complex (DISC). In DISC, FADD interacts with the DED domain of caspase-8 through its death effect domain (DED) to recruit and activate caspase-8. Activated caspase-8, as the initial caspase, can further cut and activate downstream effector caspases, such as caspase-3, caspase-6 and caspase-7, etc. These effector caspases trigger the cascade reaction of apoptosis by cutting various key substrates in cells. Eventually, it leads to nerve cell apoptosis (149, 150).

In addition, IL-1β can induce neuronal apoptosis by activating the NF-κB signaling pathway within nerve cells. Il-1β binds to the IL-1 receptor (IL-1R) on the surface of nerve cells, triggering a conformational change of the receptor and further recruitment of myeloid differentiation factor 88 (MyD88) (151). MyD88 interacts with IL-1 receptor-associated kinase (IRAK) through its death domain to activate IRAK. Activated IRAK further phosphorylates and activates tumor necrosis factor receptor-associated factor 6 (TRAF6). TRAF6 activates TAK1 by binding to transforming growth factor-β-activated kinase 1 (TAK1) (152). TAK1 phosphorylates and activates the IκB kinase (IKK) complex, which phosphorylates the IκB protein, resulting in the degradation of the IκB protein, resulting in the release of NF-κB. After entering the nucleus, NF-κB binds to the κB site in the promoter region of the target gene, promoting the expression of a series of pro-inflammatory genes and apoptosis-related genes, such as TNF-α, IL-6 and apoptotic protease activator 1 (Apaf-1), and the expression products of these genes further mediate apoptosis of nerve cells (153). In addition, IL-1β can induce nerve cell apoptosis through mitochondrial pathway. After stimulation of nerve cells by IL-1β, the mitochondrial membrane potential decreases and the mitochondrial permeability transition pore (MPTP) opens, allowing the mitochondria to release Cyt C into the cytoplasm. Cyt C combines with Apaf-1 and dATP to form apoptotic bodies, which recruit and activate caspase-9, and the activated caspase-9 further activates downstream effector caspases, such as caspase-3, leading to apoptosis (154).

### 5.3 Damage the blood-spinal barrier

Blood-spinal barrier is an important barrier between the blood circulatory system of the spinal cord and the spinal cord tissue, which is composed of vascular endothelial cells, basal membrane, astrocytes and foot process (155). Blood-spinal barrier can effectively maintain the stability of the environment in the spinal cord tissue, prevent harmful substances from entering the spinal cord tissue, and play a crucial protective role in the normal physiological function of the spinal cord (156). A breach of the blood-spinal barrier can lead to a number of serious consequences. The exudation of plasma proteins and inflammatory cells can cause edema of the spinal cord tissue, which further compresses the spinal cord tissue and aggravates neurological dysfunction. The entry of harmful substances will cause direct damage to nerve cells and glial cells, leading to apoptosis and necrosis. Infiltration of inflammatory cells will further activate the inflammatory response, forming a vicious cycle and continuously aggravating the degree of SCI (157).

Inflammatory mediators play a key role in the breakdown of the blood-spinal barrier after SCI. In the early stages of SCI, damaged tissues rapidly release large amounts of TNF-α and IL-1β. TNF-α can bind to TNF receptors on the surface of vascular endothelial cells and activate intracellular MAPK and NF-κB signaling pathways. Activation of these signaling pathways leads to the expression and release of various adhesion molecules by endothelial cells, such as intercellular adhesion molecule-1 (ICAM-1) and vascular cell adhesion molecule-1 (VCAM-1), etc. These adhesion molecules can promote the adhesion of white blood cells to vascular endothelial cells, making it easier for white blood cells to enter the spinal cord through vascular endothelial cells. This increases the permeability of the blood-spinal barrier (158). In addition, IL-1β can also induce vascular endothelial cells to produce inflammatory mediators such as NO and prostaglandin, which can lead to vasodilation and increased permeability, make plasma proteins and inflammatory cells more easily leak into the spinal cord tissue, and destroy the integrity of the blood-spinal barrier (159).

In addition, inflammatory mediators can also affect the tight junction protein of the blood-spinal barrier, which is an important part of maintaining the structure and function of the blood-spinal barrier. Under the action of inflammatory mediators, the expression and distribution of these tight-linking proteins are altered. TNF-α and IL-1β can change the phosphorylation level of tight junctions by activating intracellular signaling pathways, resulting in looser structures of tight junctions and larger gaps, thus increasing the permeability of the blood-spinal barrier (160).

### 5.4 Promote glial scar formation

Although the formation of glial scar is a self-protective response of the body to SCI to a certain extent, which can isolate the injured site and prevent the further spread of inflammation, excessive glial scar formation has become a huge obstacle to nerve regeneration (161).

**Figure 3:**
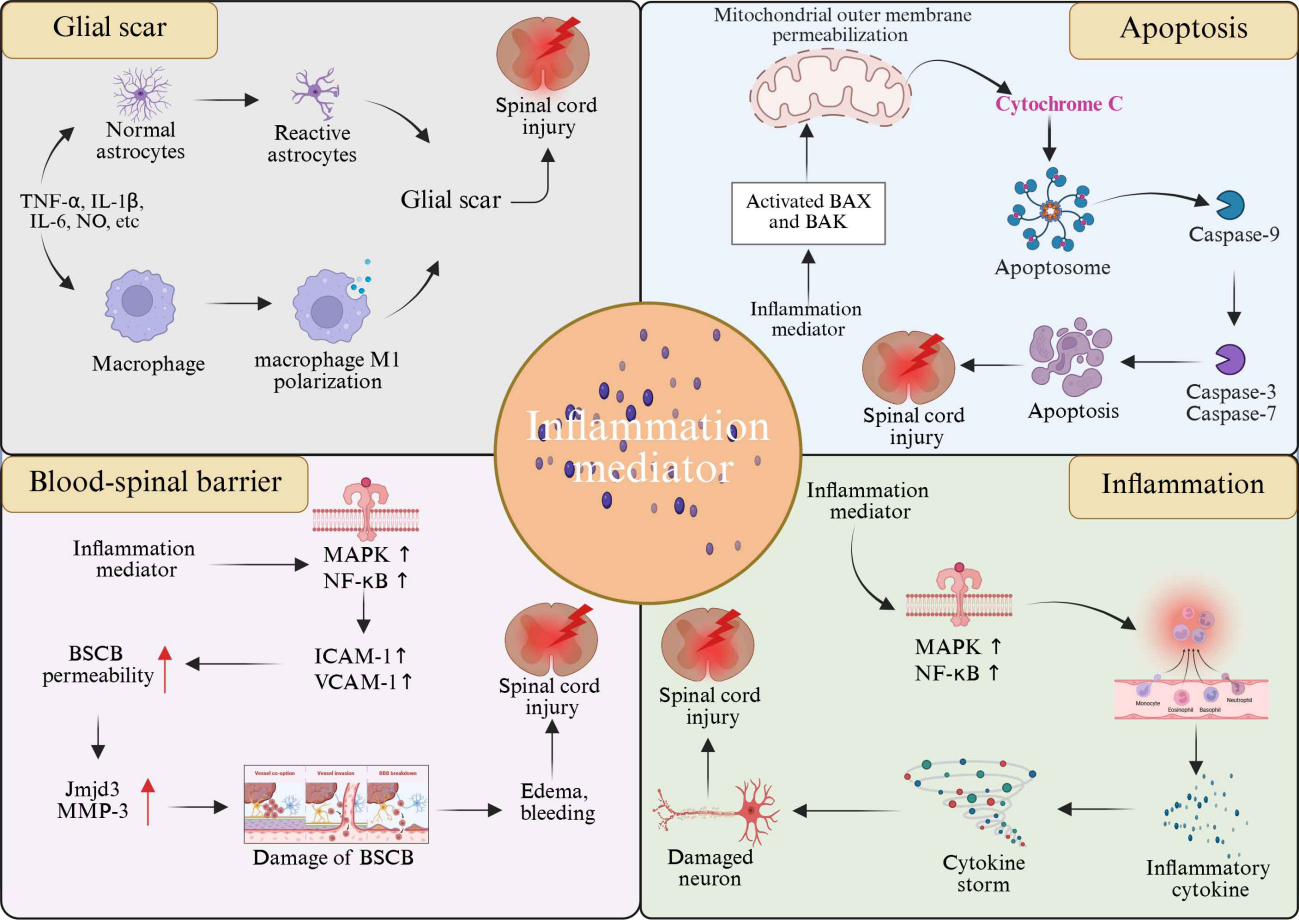
Role of inflammatory mediators in SCI progression. Inflammatory mediators have been implicated in the pathologic progression of SCI, including mediating inflammatory responses, apoptosis, glial scarring, and breakdown of the blood-spinal barrier.

After SCI, the massive release of inflammatory mediators strongly stimulates astrocytes, causing them to rapidly activate and proliferate in large quantities (162). Among them, pro-inflammatory cytokines such as TNF-α and IL-1β play a key role in glial scar formation. TNF-α closely binds to specific receptors on the surface of astrocytes and activates a series of complex intracellular signaling pathways, such as the NF-κB signaling pathway. Activated NF-κB enters the nucleus, interacts with specific DNA sequences, initiates transcription of a series of genes associated with glial scarring, and promotes accelerated proliferation of astrocytes (163). In addition, IL-1β also has a powerful stimulating effect, which can further enhance the activity of astrocytes and promote their proliferation and differentiation by activating the MAPK signaling pathway in astrocytes. Under the continuous stimulation of these inflammatory mediators, astrocytes continue to prolifate and gradually gather at the injured site to form immature glial scars. Over time, glial scars mature, forming a physical and chemical barrier that impedes the growth and extension of nerve axons (164) (**Figure 3**).

Although inflammatory mediators mainly drive secondary nerve injury in SCI, the latest research shows that some mediators have key functions of neuroprotection, repair promotion and immune homeostasis regulation under specific spatiotemporal conditions. Pro-inflammatory mediators exhibit a “double-edged sword” characteristic of concentration and time dependence: Low concentrations of TNF-α can activate the neuroprotective NF-κB pathway and stimulate the proliferation of oligodendrocyte precursor cells, supporting myelin regeneration (165). Moderate IL-1β can induce the release of neurotrophic factors (such as BDNF) and promote the formation of protective glial scars. Neuron-derived IL-6 enhances neuronal survival and axon regeneration ability through the sIL-6R/gp130 signaling axis (166). Anti-inflammatory and restorative mediators play a dominant protective role: IL-10 strongly inhibits the storm of pro-inflammatory factors, drives the polarization of M2-type macrophages/microglia and secretes restorative factors (TGF-β, IGF-1) (167). TGF-β not only inhibits excessive inflammation but also reshapes the extracellular matrix to provide a scaffold for nerve repair. IL-4/IL-13 promotes the release of neurotrophic factors (GDNF, NGF) and myelin regeneration by inducing M2 polarization (168, 169). Lipid-derived Resolvins and other inflammatory resolution mediators (SPMs) actively terminate the inflammatory response, enhance the clearance of apoptotic cells and protect the mitochondrial function of neurons. In addition, chemokines are also involved in repair regulation. For instance, CXCL12 guides neural stem cells to migrate to the injury area and promotes angiogenesis, while low levels of CCL2 can recruit regulatory T cells (Tregs) to inhibit excessive amplification of inflammation (170).

## 6 Signaling pathways involved in regulating inflammatory mediators

### 6.1 Toll-like receptor pathway

Toll-like receptor (TLR) is a pattern recognition receptor encoded by the germline (171). There are many members of the TLR family, among which TLR4 has been extensively studied. TLR pathway plays a core role in the inflammatory response after SCI, precisely regulating the release of inflammatory mediators and the process of inflammatory response (172). In the context of SCI, the role of TLR4 is particularly critical. When the spinal cord is injured, a large number of endogenous danger signal molecules will be released at the injured site, such as heat shock protein and high mobility group protein B1 (HMGB1), which can activate TLR4 (173). After TLR4 successfully binds to the ligand, it rapidly recruits MyD88 to activate interleukin-1 receptor-associated kinase (IRAK). Activated IRAK is phosphorylated and becomes more active, interacting with tumor necrosis factor receptor-associated Factor 6 (TRAF6) to form a tight signaling complex. The formation of this complex signals a critical phase of signaling, which activates the downstream transforming growth factor-beta-activated kinase 1 (TAK1). TAK1 activates the NF-κB signaling pathway by activating NF-κB-induced kinase (NIK), which translocations from cytoplasm to the nucleus and drives a series of pro-inflammatory gene transcription, leading to the production and release of inflammatory cytokines such as TNF-α, IL-2, IL-6 and INF-γ (153). The large amount of expression and release of these inflammatory mediators can aggravate the degree of inflammatory response (174).

### 6.2 NF-κB pathway

NF-κB is considered to be a central transcription factor of inflammatory mediators and plays a crucial role in inflammation (175). After SCI, a variety of inflammatory mediators are released at the injury site. These inflammatory mediators activate tumor necrosis factor receptor-associated factor 2 (TRAF2), and TRAF2 activates the IKK complex composed of IKKα, IKkβ and regulatory subunit NEMO. IKKβ plays a key role in the activation of the NF-κB pathway. Activated IKKβ phosphorylates IκB, resulting in ubiquitination modification of IκB, which is then recognized and degraded by the proteasome. When the NF-κB pathway is activated, nuclear translocations occur, from cytoplasmic translocations to specific DNA sequences in the nucleus, initiating the transcription process of a series of pro-inflammatory genes. These pro-inflammatory genes include genes encoding inflammatory mediators such as TNF-α, IL-1β and IL-6, as well as related genes such as inducible nitric oxide synthase (iNOS) (176). With the transcription and translation of these genes, a large number of inflammatory mediators are synthesized and released outside the cell, further exacerbating the degree of inflammatory response.

### 6.3 MAPK pathway

MAPK pathway is a highly conserved signaling pathway, which mainly includes three major branches: extracellular signal-regulated kinase (ERK), c-Jun amino terminal kinase (JNK) and p38 MAPK (177, 178). MAPK pathway is a very important pathway in the body, which is involved in a series of cellular responses triggered by environmental and developmental signal transduction (179). Studies have shown that after the occurrence of SCI, a variety of inflammatory mediators can act as stimulus signals to activate MAPK pathways. The activated MAPK pathway plays a key role in the regulation of inflammatory response after injury, connecting various stimulus signals on the cell surface with the regulation mechanism of intracellular inflammatory mediators (180).

Activated p38 MAPK rapidly phosphorylates a series of downstream transcription factors, such as activating transcription factor 2 (ATF2), c-Jun, etc. These phosphorylated transcription factors can quickly enter the nucleus and bind to specific DNA sequences, thus initiating the transcription process of a series of inflammatory mediators (181). Studies have shown that the activation of p38 MAPK can promote the expression and release of pro-inflammatory cytokines such as TNF-α, IL-1β and IL-6, further aggravating the degree of inflammatory response (182). In the SCI *in vivo* model, the administration of specific inhibitors of p38 MAPK, such as SB203580, can significantly reduce the level of inflammatory mediators and alleviate the inflammatory injury of spinal cord tissue and neurological function deficit (183).

**Figure 4:**
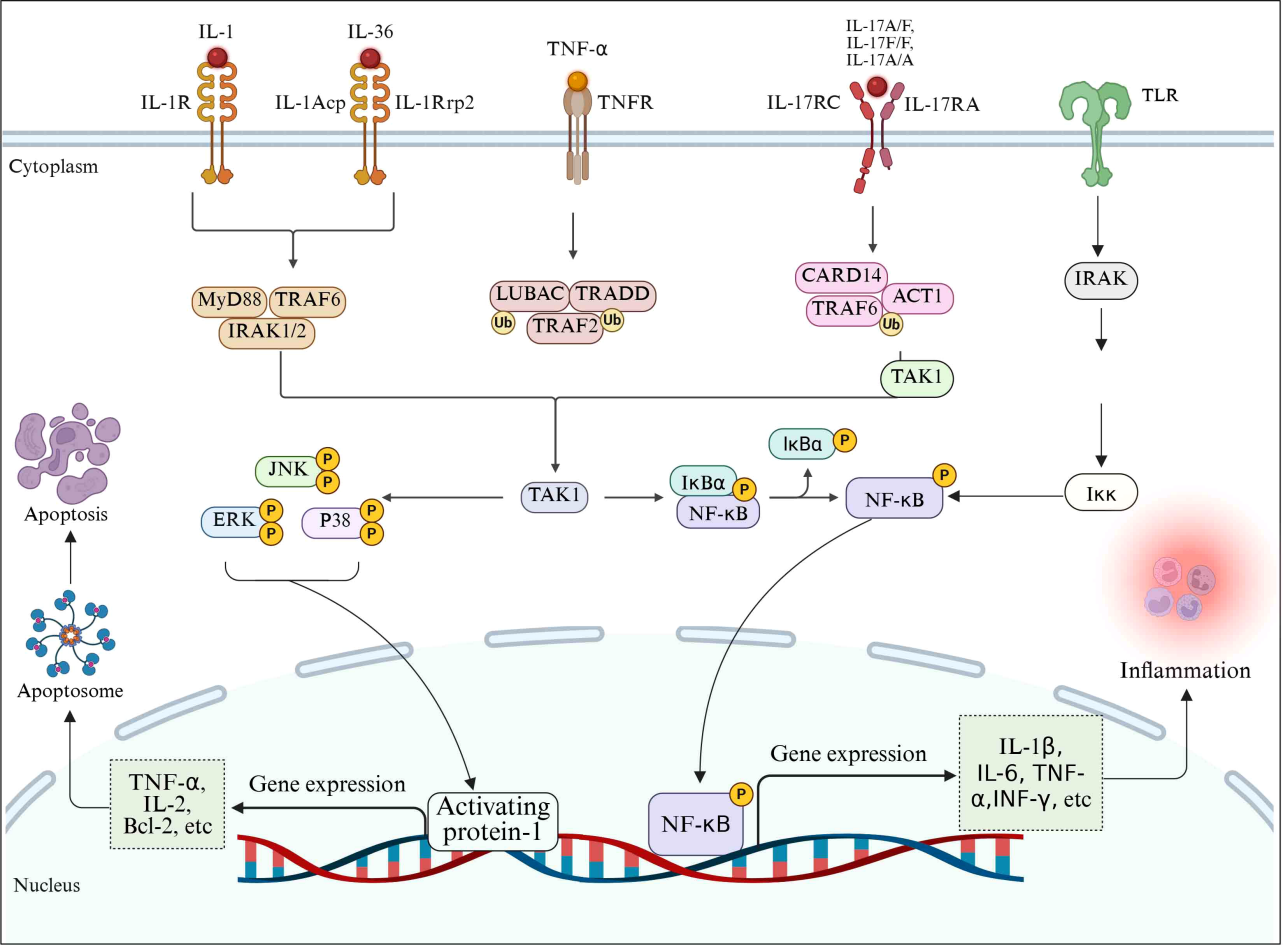
Schematic diagram of signaling pathways involved in the regulation of inflammatory mediators after SCI.

JNK also plays an important role in the inflammatory response after SCI. JNK promotes the release of inflammatory mediators by activating downstream transcription factors (184). For example, JNK can activate the NF-κB signaling pathway, further enhancing the expression and release of inflammatory mediators. *In vitro* cell experiments, inhibition of JNK activity can reduce the secretion of inflammatory mediators and alleviate the damage of inflammation to cells (185). In addition, JNK activated after SCI can promote neuronal apoptosis by regulating the expression of apoptosis related proteins (186) (**Figure 4**).

After SCI, TLR4 acts as the core pattern recognition receptor, initiating a MyD88-dependent signal cascade by recognizing endogenous danger signals (such as HMGB1, heat shock proteins): Activate the IRAK-TRAF6 complex and drive the TAK1/NF-κB pathway, inducing the transcriptional release of pro-inflammatory factors (TNF-α, IL-1β, IL-6, IFN-γ) (187). Meanwhile, NF-κB, as an inflammatory transcription center, is activated by mediators such as TNF-α/IL-1β through the TRAF2-IKK pathway, and achieves nuclear translocation by degrading IκB, further amplifying the expression of genes such as TNF-α, IL-6 and iNOS (188). The MAPK pathway (especially p38 and JNK as the core) integrates injury signals, promotes the release of inflammatory mediators by phosphorylating transcription factors such as ATF2/c-Jun, and aggravates inflammation and nerve injury by activating NF-κB and apoptotic pathways (189). The three pathways form a cascading and mutually feedable positive feedback loop, jointly driving the inflammatory storm, neuronal apoptosis and secondary damage. The key nodes (such as TLR4, IKK, p38/JNK) have become potential therapeutic targets for intervening in the malignant process of SCI inflammation.

## 7 The prospect of treating SCI for inflammation

### 7.1 Chemical synthesis antagonist

TNF-α inhibitors and IL-1 inhibitors have been shown to control and improve the inflammatory microenvironment in SCI (190–192). TNF inhibitors such as infliximab and Adalimumab have been shown to reduce inflammatory responses in SCI (193). Infliximab is a monoclonal antibody that binds to and neutralizes TNF-α, thereby rendering TNF-α biologically inactive (194). Studies have found that infliximab treatment of SCI rats and rabbits can significantly inhibit vascular response and reduce neuron loss (195, 196). Adamylizumab (ADM) is a humanized anti-tumor necrosis factor (TNF) antibody. In a rat model of SCI, ADM combined with erythropoietin can inhibit microglia M1 polarization mediated neuroinflammation and apoptosis, thereby improving the recovery of SCI (197). IL-1 receptor antagonist (IL-1Ra) is an endogenous competitive antagonist that binds to IL-1 receptor (IL-1R) and blocks the transmission of inflammatory signals, thereby inhibiting subsequent downstream pro-inflammatory events (198). Studies have found that the administration of peripheral IL-1Ra after SCI can weaken the acute phase response and reduce the infiltration of immune cells at the injury site, thus promoting the recovery of nerve function (199). COX-2 is also a target for controlling and improving the inflammatory microenvironment because it regulates the synthesis of PGE2 in the inflammatory microenvironment (200). In the SCI rat model, peritoneal injection of COX-2 inhibitors can reduce the inflammatory response of rats by inhibiting neutrophil infiltration and reducing prostaglandin and free radical synthesis (201).

### 7.2 MSC therapy

In recent years, a variety of cellular therapies have been developed for the repair and regeneration of nerve function after SCI (202, 203). Among all candidate cells, mesenchymal stem cells (MSCs) have the greatest potential for regeneration of nerve function after injury, due to their autotransplantation ability (204). MSC is a pluripotent stem cell derived from mesoderm, which has the ability of self-renewal and multidirectional differentiation. MSCS are regarded as pluripotent “seed cells” that inhibit local inflammation, apoptosis, and stimulate the regeneration and differentiation of resident tissue progenitor cells by secreting soluble growth and trophic factors (205, 206). Transplanted MSCs can reduce the inflammatory response at the injured site by secreting anti-inflammatory cytokines and inhibiting pro-inflammatory cytokines (such as TNF, IFN-γ, and IL-6), and play a crucial role in promoting tissue repair (207, 208). In a mouse model of contusion SCI, transplantation of neural stem cells (NSC) to the injury site reduced neutrophils and regulated macrophage activation by inhibiting M1 macrophage activation. NSC reduces mRNA levels of inflammatory cytokines, including TNF-α, IL-1β, IL-6, and IL-12. NSC can also inhibit the activation of bone marine-derived macrophages, reduce the release of cytokines such as TNF-α and IL-1β, and improve functional recovery after SCI (209). Although the benefits and promising results of MSC-based therapies have been observed, the mechanisms are still not clearly elucidated in animal experiments, and most clinical studies are case reports with limited sample sizes.

### 7.3 Hormonal therapy

Hormone therapy is a therapeutic method to regulate SCI inflammation by applying estrogen and progesterone (210, 211). Estrogen has been shown to have a neuroprotective effect due to its anti-inflammatory effect, and the gene expression of TNF-α and its downstream cytokine iNOS can be significantly reduced after estrogen therapy in rats with SCI (212). Progesterone is also an anti-inflammatory hormone that inhibits inflammatory cytokines. In SCI rat models, progesterone treatment can significantly reduce the expression of TNF-α and iNOS and down-regulate inflammatory cytokines (such as iNOS, MCP-1 and IL-1β), thus inhibiting the post-injury inflammatory response (210) (**Table 2**).

**Table 2:** Potential regulatory modes and regulators of inflammation mediator in SCI treatment.

Regulation modes/regulator	Mechanisms	References
Chemical synthesis antagonist		
Infliximab	Binds and neutralizes TNF-α, rendering TNF-α biologically inactive→Inhibiting vascular response and reducing neuronal loss	(195, 196)
Adalimumab	Inhibition of microglia M1 polarization→Improve the recovery of SCI	(197)
COX-2 inhibitor	Binds to IL-1 receptor and blocks the transmission of inflammatory signals→Inhibits neutrophil infiltration, and reduces the synthesis of prostaglandins and free radicals	(201)
MSC therapy		
ADSCs	Inhibit M1 macrophage activation→Reduce the inflammatory cytokine mRNA levels→Improve the functional recovery after SCI	(213)
BMSCs	Inhibit M1 macrophage activation→Reduce the inflammatory cytokine mRNA levels→Improve the functional recovery after SCI	(209)
Hormonal therapy		
Progesterone	Reduce the expression of TNF-α and iNOS and inflammatory cytokines→inhibited the inflammatory response	(210)
Estrogen	Reduce the expression of TNF-α and iNOS and inflammatory cytokines→Inhibited the inflammatory response	(212)

ADSCs, Adipose Derived Stem Cells; BMSCs, Bone marrow mesenchymal stem cells.

## 8 The current limitations of research and future research directions

### 8.1 The limitations of the current research

Although this study has achieved certain results in the research field of inflammatory mediators and SCI, there are still some deficiencies. In terms of the depth and breadth of research, the regulatory network of inflammatory mediators in spinal cord injury is extremely complex, involving the interaction of multiple cell types and signaling pathways. Although current studies have explored some key inflammatory mediators and signaling pathways, there is still a lack of comprehensive understanding of the entire regulatory network. The synergistic or antagonistic effects among different inflammatory mediators, as well as their dynamic change patterns at different pathological stages, still await further in-depth research. In the research of treatment strategies, although various treatment strategies based on the regulation of inflammatory mediators have been proposed, these strategies face many challenges in the process of clinical transformation. In terms of drug therapy, the research and development of emerging drugs is still in its infancy, and issues such as the safety, efficacy and administration routes of the drugs have not been completely resolved. The mechanisms of action of physical therapy and cell therapy still need further in-depth research to optimize treatment plans and improve therapeutic effects. In addition, current research mainly focuses on cell and animal experiments, lacking large-scale clinical trials for verification, which also limits the promotion and application of research results.

### 8.2 Future perspectives

The research on inflammatory mediators in SCI holds broad prospects. Future studies can be carried out in the following directions. By delving deeply into the interaction networks among inflammatory mediators and leveraging techniques such as systems biology and bioinformatics, a regulatory network model of inflammatory mediators is constructed to comprehensively reveal their synergistic and antagonistic relationships, providing a theoretical basis for the development of multi-target therapeutic strategies. By delving deeply into the interaction networks among inflammatory mediators and leveraging techniques such as systems biology and bioinformatics, a regulatory network model of inflammatory mediators is constructed to comprehensively reveal their synergistic and antagonistic relationships, providing a theoretical basis for the development of multi-target therapeutic strategies. Continue to develop new treatment methods and drugs, and target the key action sites of inflammatory mediators to develop safer and more effective treatment approaches. By integrating emerging technologies such as gene therapy, nanotechnology and biomaterials, the precision and effectiveness of treatment can be enhanced. Continue to develop new treatment methods and drugs, and target the key action sites of inflammatory mediators to develop safer and more effective treatment approaches. By integrating emerging technologies such as gene therapy, nanotechnology and biomaterials, the precision and effectiveness of treatment can be enhanced.

## 9 Conclusion

Spinal cord injury is a common neurological disorder that can cause disability and negatively affect quality of life. Inflammation is a related process in the pathophysiology of SCI and is regarded as an important factor in the occurrence and development of SCI. Various inflammatory mediators play a crucial role in SCI and the inflammatory microenvironment. The research on the regulatory role of inflammatory mediators in SCI has important theoretical and clinical significance. They lead to further damage of the spinal cord by regulating pathological processes such as inflammatory response, apoptosis and glial scar formation in SCI. Targeted inhibition of inflammatory mediators can effectively suppress the above pathological processes, thereby alleviating SCI symptoms and improving prognosis. Although there are currently some deficiencies, through continuous in-depth research and exploration, it is expected to provide new ideas and bring new breakthroughs for future SCI treatment based on inflammatory mediators, thereby improving the prognosis and quality of life of patients.
